# Supplementary material for: Taxonomic and Metagenomic Survey of a Peat-Based Straw Degrading Biofertilizer
Source: Microorganisms. 2025 Dec 12;13(12):2830. doi: 10.3390/microorganisms13122830 (PMC12735889; doi:10.3390/microorganisms13122830)
Supplement: Supplementary file 1 [file microorganisms-13-02830-s001.zip › bags_figure_supplement.pdf]

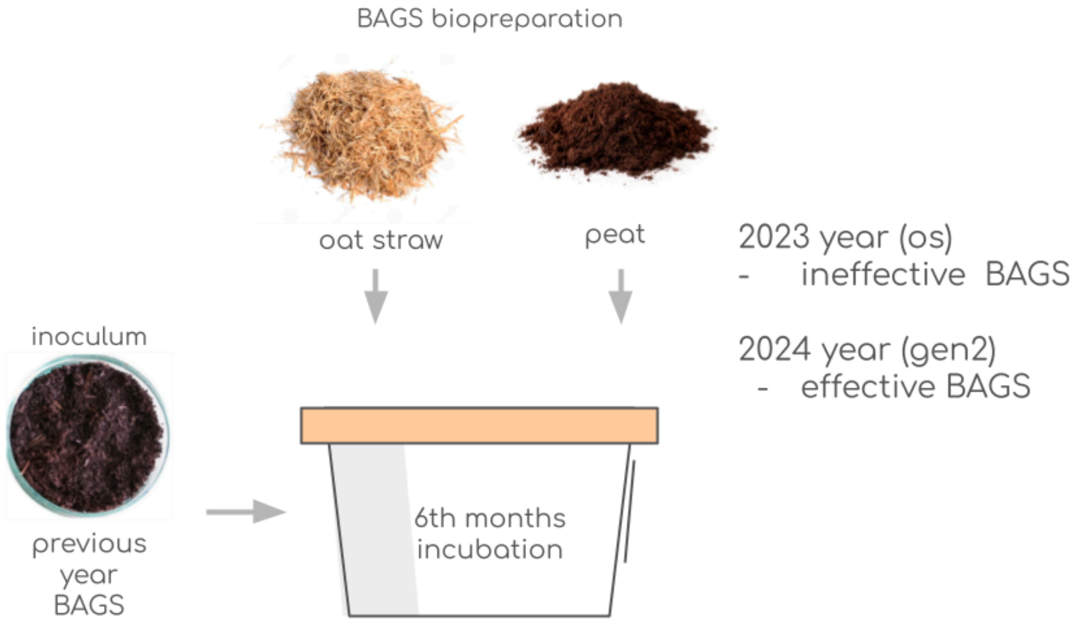

Figure S1. The scheme of BAGS incubation.

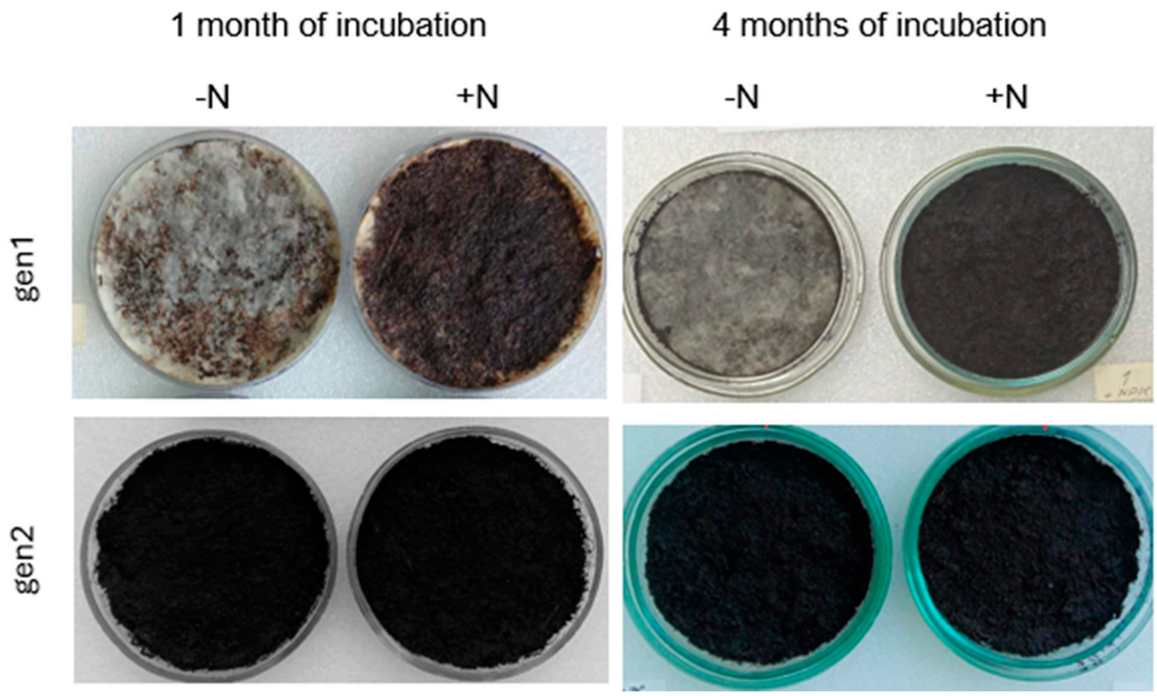

Figure S2. The cellulolytic activity of BAGS preparation. The activity is visualized by a degree of paper filter maceration. (-N) without stimulation by additional nitrogen, (+N) with stimulation by additional nitrogen. Upper row – gen1 BAGS from 2023, lower row – gen2 BAGS from 2024. Left column – preparation after 1 month of composting, right column – after 4 months of composting. Incubation with paper filter in all cases was for 2 months.

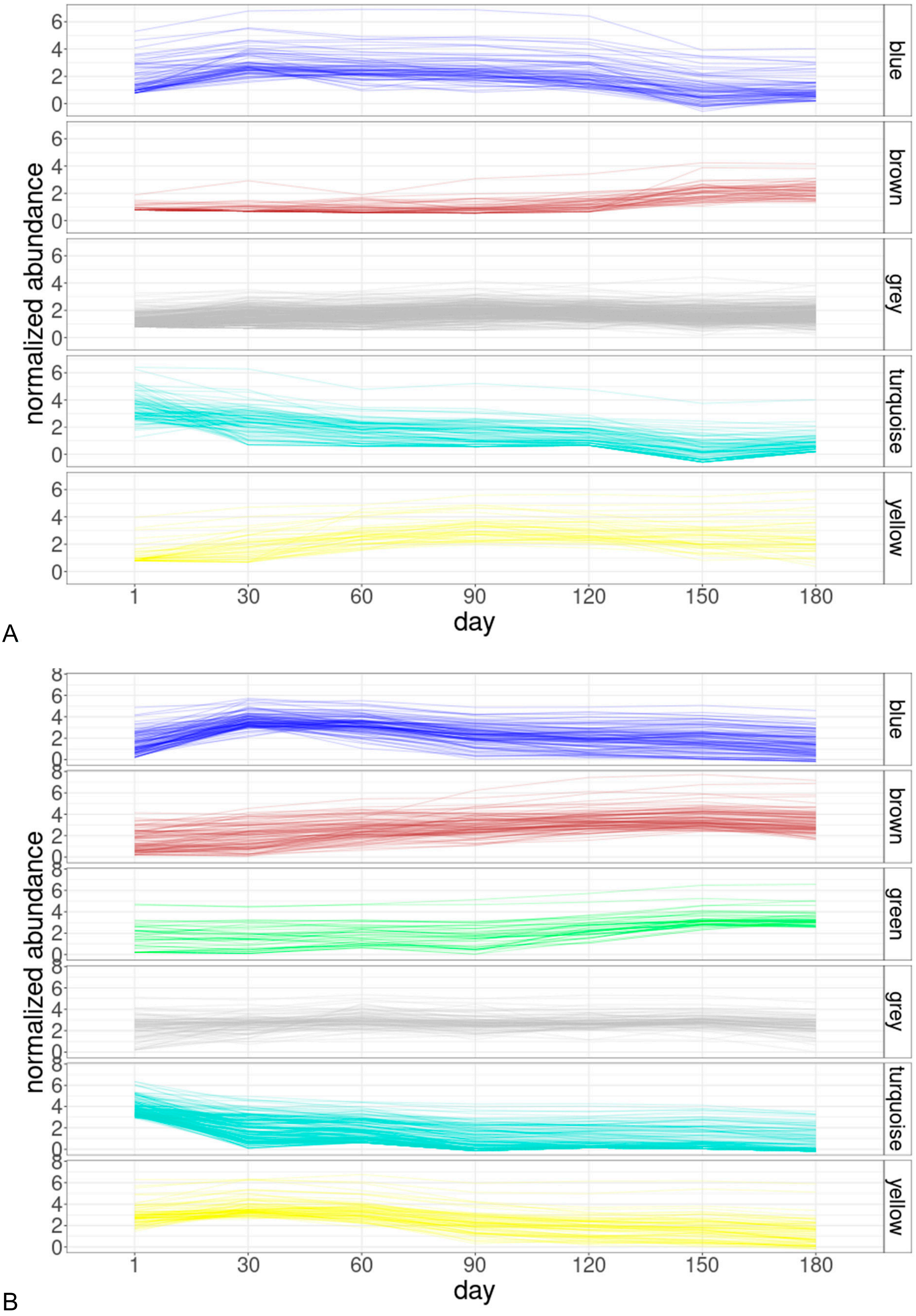

Figure S3. The network analysis (WGCNA) of ASVs coexpression in BAGS composting process. The normalized abundance of ASV clusters in gen1 BAGS (A) and gen2 BAGS (B).

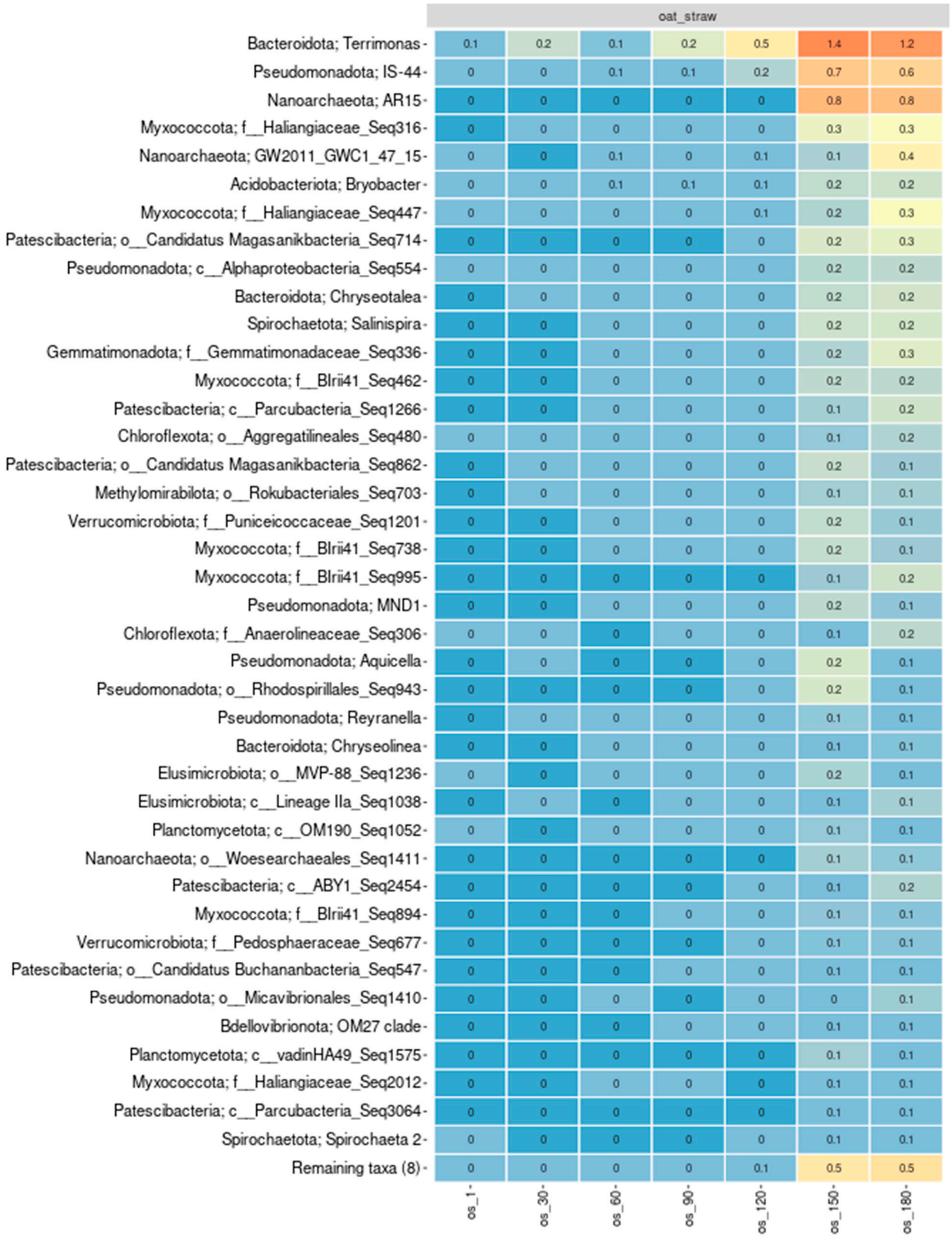

Figure S4. The heatmap of brown cluster of ASVs in gen1 BAGS.

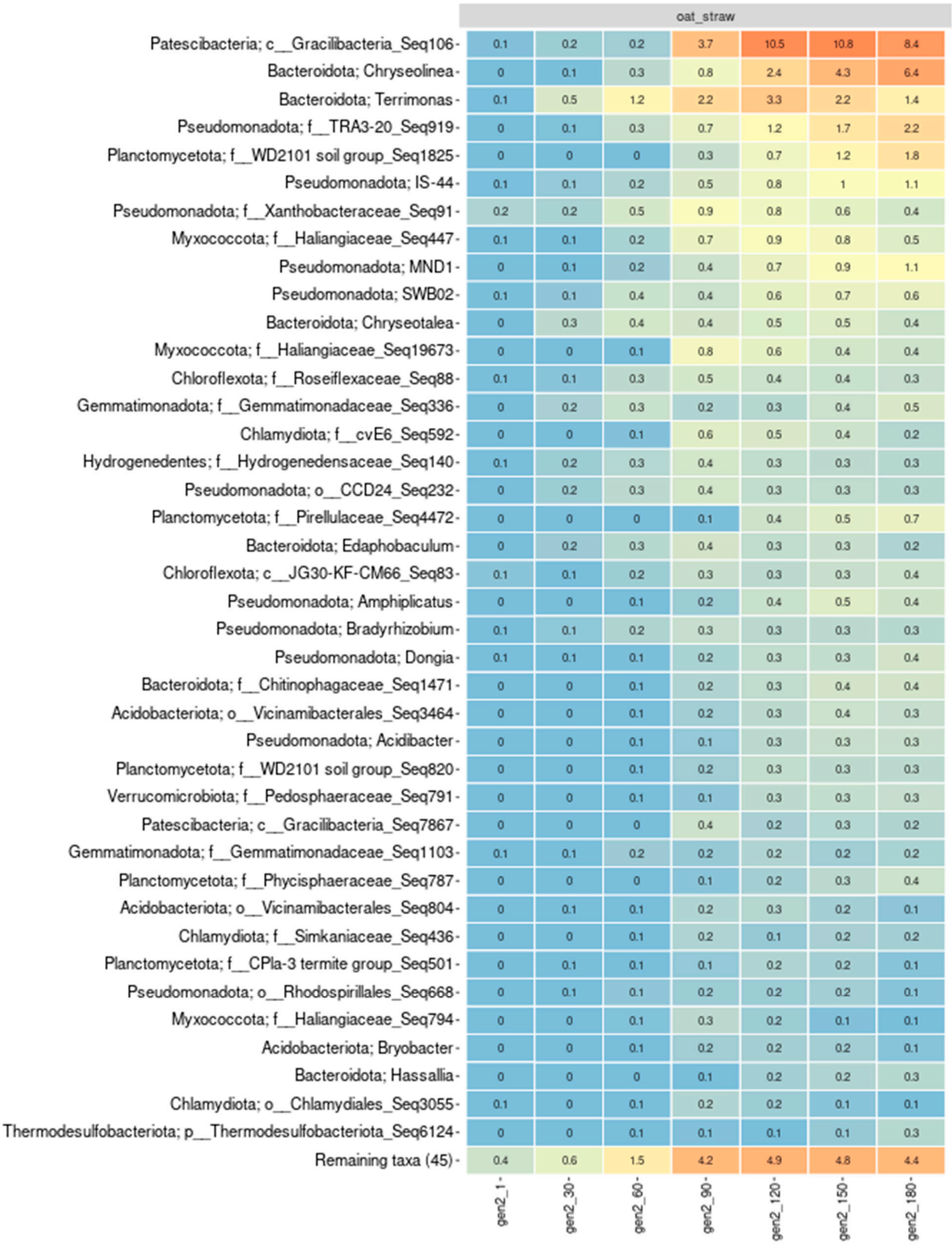

Figure S5. The heatmap of brown cluster of ASVs in gen2 BAGS.

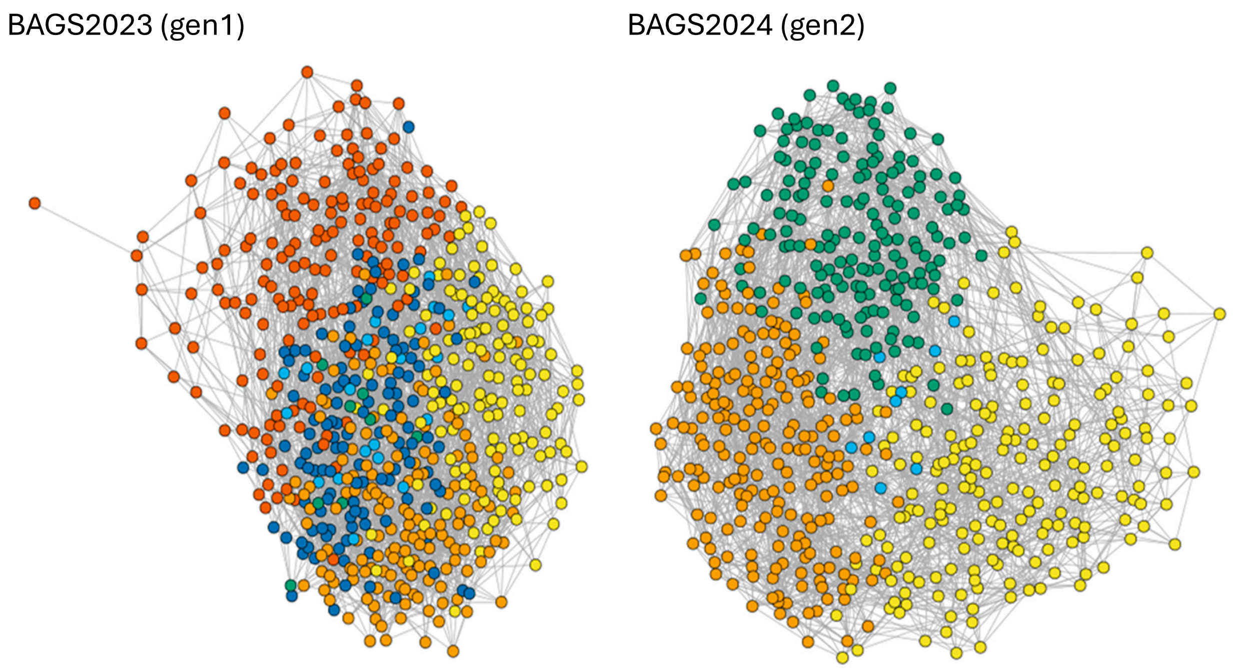

Figure S6. The cooccurrence network for gen1 BAGS (left) and gen2 BAGS (right).

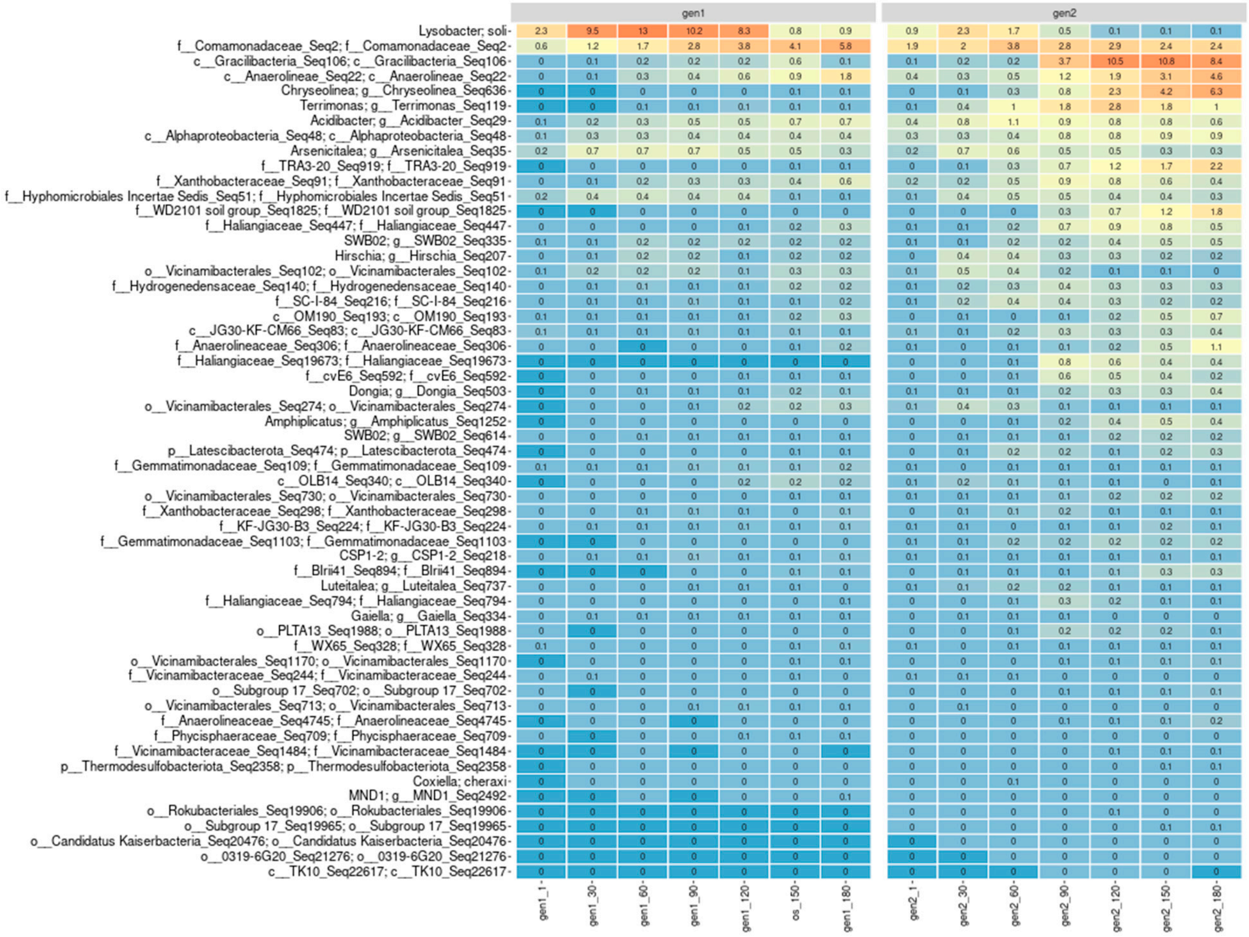

Figure S7. The heatmap of ASVs, correlating with MAGs, isolated from the metagenome of the mature phase of the effective BAGS.
